# Case of Hemiplegia Consequent on Tying the Common Carotid

**Published:** 1843-09-16

**Authors:** O. Fairfax

**Affiliations:** Alexandria, (D. C.)


### CASE OF HEMIPLEGIA, CONSEQUENT ON TYING THE COMMON CAROTID.

BY O. FAIRFAX, M. D.

To the Editor of the Medical Examiner.

*Sir*,—Observing in a late number of the Medical Examiner, an instance of hemiplegia, consequent on tying the common carotid, and believing it to be an unusual effect of the operation, and an additional and striking exemplification of the fact that opposite conditions of the brain as regards its supply of blood, may result in the production of some symptoms in common, I am induced to send you the following similar case.

On the 18th of July, 1842, in presence of Drs. Murphy, Powell and Richards, of this place, I applied a ligature to the left common carotid, an inch above the clavicle, and my patient became hemiplegiac, as regards the limbs of the right side, but the face was not affected. The hemiplegia may have occurred at the moment of tying the ligature, but was not remarked by the attendants until an hour or more after the operation. The patient, who was a middle aged lady, already much reduced by chronic disease of the lungs, remained faint and hemiplegiac to her death, which occurred after five days, apparently from exhaustion. Her mental faculties continued perfect to the last hour.

*Alexandria, (D. C.)* Aug. 31, 1843.

